# Performance of Prognostication Scores for Mortality in Injured Patients in Rwanda

**DOI:** 10.5811/westjem.2020.10.48434

**Published:** 2021-01-22

**Authors:** Oliver Y. Tang, Catalina González Marqués, Vincent Ndebwanimana, Chantal Uwamahoro, Doris Uwamahoro, Zachary W. Lipsman, Sonya Naganathan, Naz Karim, Menelas Nkeshimana, Adam C. Levine, Andrew Stephen, Adam R. Aluisio

**Affiliations:** * Brown University Warren Alpert Medical School, Department, Providence, Rhode Island; † Brown University Warren Alpert Medical School, Department of Emergency Medicine, Providence, Rhode Island; ‡ University of Rwanda, Department of Anesthesia, Emergency Medicine, and Critical Care, Kigali, Rwanda; § Centre Hospitalier Universitaire de Kigali, Department of Accident & Emergency, Kigali, Rwanda; ¶ Kaiser Permanente, GSAA, San Leandro & Fremont Medical Centers, San Leandro, California; || Brown University Warren Alpert Medical School, Department of Surgery, Providence, Rhode Island

## Abstract

**Introduction:**

While trauma prognostication and triage scores have been designed for use in lower-resourced healthcare settings specifically, the comparative clinical performance between trauma-specific and general triage scores for risk-stratifying injured patients in such settings is not well understood. This study evaluated the Kampala Trauma Score (KTS), Revised Trauma Score (RTS), and Triage Early Warning Score (TEWS) for accuracy in predicting mortality among injured patients seeking emergency department (ED) care at the Centre Hospitalier Universitaire de Kigali (CHUK) in Rwanda.

**Methods:**

A retrospective, randomly sampled cohort of ED patients presenting with injury was accrued from August 2015–July 2016. Primary outcome was 14-day mortality and secondary outcome was overall facility-based mortality. We evaluated summary statistics of the cohort. Bootstrap regression models were used to compare areas under receiver operating curves (AUC) with associated 95% confidence intervals (CI).

**Results:**

Among 617 cases, the median age was 32 years and 73.5% were male. The most frequent mechanism of injury was road traffic incident (56.2%). Predominant anatomical regions of injury were craniofacial (39.3%) and lower extremities (38.7%), and the most common injury types were fracture (46.0%) and contusion (12.0%). Fourteen-day mortality was 2.6% and overall facility-based mortality was 3.4%. For 14-day mortality, TEWS had the highest accuracy (AUC = 0.88, 95% CI, 0.76–1.00), followed by RTS (AUC = 0.73, 95% CI, 0.55–0.92), and then KTS (AUC = 0.65, 95% CI, 0.47–0.84). Similarly, for facility-based mortality, TEWS (AUC = 0.89, 95% CI, 0.79–0.98) had greater accuracy than RTS (AUC = 0.76, 95% CI, 0.61–0.91) and KTS (AUC = 0.68, 95% CI, 0.53–0.83). On pairwise comparisons, RTS had greater prognostic accuracy than KTS for 14-day mortality (P = 0.011) and TEWS had greater accuracy than KTS for overall (P = 0.007) mortality. However, TEWS and RTS accuracy were not significantly different for 14-day mortality (P = 0.864) or facility-based mortality (P = 0.101).

**Conclusion:**

In this cohort of emergently injured patients in Rwanda, the TEWS demonstrated the greatest accuracy for predicting mortality outcomes, with no significant discriminatory benefit found in the use of the trauma-specific RTS or KTS instruments, suggesting that the TEWS is the most clinically useful approach in the setting studied and likely in other similar ED environments.

## INTRODUCTION

The impact of injury disproportionately falls on the health systems of low- and middle-income countries (LMIC). While the World Health Organization has estimated that 16% of global disease burden is due to injury,^1^ approximately 90% of deaths and disability-adjusted life years lost due to injury occur in LMICs.^2^,^3^ Commonly required injury care in LMICs is challenged by limited material resources and healthcare personnel.^4^ In particular, although sub-Saharan Africa has a high burden of injury, the region has just 3% of the world’s healthcare workers and less than 1% of healthcare resources.^5^

Triage systems are an important method to assist in addressing health barriers as they can facilitate the prompt identification of patients with the most urgent needs and highest risks.^6^,^7^ Prior research has demonstrated that triage systems used in acute care settings in LMICs are associated with reduced time to treatment and mortality.^8^,^9^ Trauma prognostication scores, which are designed to stratify patient severity and predict mortality, have the potential to enhance triage for injured patients.^10^ Although the Revised Trauma Score (RTS) has been used in high-income countries (HIC),^11^ this metric and other scores initially developed in HICs may have limited application in LMICs.^5^,^10^ Accordingly, the Kampala Trauma Score (KTS) was developed in Uganda in 1996 for use in trauma prognostication in sub-Saharan Africa specifically, and has since been validated.^3^,^5^,^10^,^12^ Several studies comparing the RTS and KTS have shown that both scores have clinical utility in risk-stratifying injury cases and predicting mortality in sub-Saharan Africa,^2^,^3^,^5^,^10^ but their accuracy has not been directly compared to established triage tools that are more broadly applicable to both injured and non-injured patients.

In most emergency care settings, general triage systems applicable to all types of patients presenting for care are used. As HIC triage tools have previously been shown to lack applicability in LMIC settings,^13^,^14^ the Triage Early Warning Score (TEWS) was developed as a contextually appropriate score for triage use in Africa.^15^ The TEWS is a component of the South African Triage Scale (SATS), which has been used and studied in multiple countries in sub-Saharan Africa including South Africa,^16^–^18^ Ghana,^19^ Somaliland,^20^ Malawi,^21^ and Rwanda.^14^ Although trauma prognostication scores such as the KTS and RTS have been extensively compared to each other,^2^ there are minimal data evaluating the clinical accuracy of the prolifically used TEWS as compared to trauma scores.^22^,^23^ As a result, it is unclear whether there is additional benefit conferred by the use of injury-specific scores in LMIC settings for acutely injured patients beyond the use of standard triage approaches. This study compared the accuracy of the KTS, RTS, and TEWS in predicting mortality for injured patients at the emergency department (ED) of the Centre Hospitalier Universitaire de Kigali (CHUK), a tertiary care hospital in Rwanda that has implemented use of the TEWS in standard emergency care triage practice.^14^

**Population Health Research Capsule**

What do we already know about this issue?

Trauma-specific and general triage scores can stratify injury mortality-risk, but the comparative accuracy of scores in low-resource settings is poorly understood.

What was the research question?

What is the accuracy of the Kampala Trauma Score, Revised Trauma Score, and Triage Early Warning Score (TEWS) in predicting injury mortality at a Rwandan tertiary hospital?

What was the major finding of the study?

Among injury patients, the TEWS demonstrated the highest accuracy in predicting 14-day and overall mortality.

How does this improve population health?

General triage scores like the TEWS may be the most clinically useful approach in the studied setting and trauma-specific scores may offer little additional utility.

## METHODS

### Study Setting and Population

This retrospective cohort study analyzed patients presenting to the ED of the CHUK in Kigali, Rwanda. CHUK is Rwanda’s primary national public referral hospital, with approximately 500 inpatient beds and an ED that provides continuous 24 hours a day care with access to specialty diagnostic, medical, and surgical services. The CHUK ED receives approximately 20,000 visits annually and maintains the country’s only emergency medicine (EM) residency training program.^4^,^24^ Data collection and research activities were approved by the CHUK Ethics Committee.

All patients presenting to the ED from August 2015–July 2016 were eligible for inclusion. To reduce selection bias, cases for analysis were randomly selected based on standardized methods that have been described previously.^4^,^25^,^26^ All ED cases were initially identified from an electronic hospital database using a composite patient identification index based on name, age, gender, home district, and date of service. All ED cases admitted during the study period were extracted using the index and subsequently coded using a unique identification number. Cases were sampled at random within each month of the accruement period (range: 135–165 cases per month). After cases were screened, we excluded all patients with insufficient documentation for data abstraction, those without acute injuries, and non-adults defined as those less than 15 years of age.^26^

### Data Management and Statistical Analysis

Data collected included patient demographics, prehospital care information, clinical presentation, past medical history, mechanism of injury, performance of surgical interventions following admission, and outcomes. We calculated three triage and prognostication scores for each patient following previously published formulas: the KTS^12^; RTS^27^; and TEWS (Appendix 1).^15^ A lower KTS or RTS score denotes a likely higher acuity in patient presentation. In contrast, a higher TEWS score indicates likely greater acuity. Serious injuries were identified as a traumatic pathology that would require hospital admission, with the number of serious injuries based on the sum number of anatomical regions of injuries involved as classified by the Abbreviated Injury Scale, as has been previously performed.^12^,^28^ All variables were collected using a standardized data instrument and entered into a password-protected database by protocol-trained personnel.^4^,^25^,^26^ Data procedures followed practices for high-quality chart review research.^29^ Ten percent of entries were double-entered. For double-entered records, assessment of data quality was performed by calculating inter-rater reliability (IRR) via Cohen’s kappa (κ).^30^

We conducted statistical analyses using Stata version 15 (StataCorp, College Station, TX) and R version 3.5.13 (R Foundation for Statistical Computing, Vienna, Austria). Summary statistics were calculated, with frequencies and percentages reported for categorical variables or medians with interquartile ranges (IQR) reported for continuous variables. The discriminatory capability of each score was quantified using nonparametric receiver operating curve (ROC) analysis, with bootstrapping (5000 iterations) performed to calculate 95% confidence intervals (CI). The primary outcome for analysis was 14-day, facility-based mortality, which included mortality during ED care and inpatient admission. The 14-day time point was chosen as it has been used in prior evaluations of prognostication scores in the East Africa context and previous data from the study setting has demonstrated that inpatient lengths of stay (LOS) at the study site for patients admitted from the ED have an LOS IQR of 2–14 days.^4^,^10^ The secondary outcome was overall facility-based mortality, which recorded patients who died before discharge, regardless of duration after presentation.

Patients discharged or transferred from the CHUK to other health facilities were assumed to have survived. Area under the curve (AUC) for scores were compared using paired bootstrap hypothesis testing.^31^ During AUC calculation for single scores, we analyzed all patients with non-missing data for the specific score of interest. For comparative analyses, patients with data for all three scores were analyzed. To evaluate for potential selection bias due to cases with missing data being excluded, we compared differences in case characteristics for cases with and without data on all three scores. Differences in case characteristics were also assessed for cases with and without data on mortality. We used Pearson’s chi-squared and Fisher’s exact tests for categorical variables, and the nonparametric Mann-Whitney test used for continuous variables.

In accordance with Bonferroni correction for multiple testing, statistical significance was maintained at *P*<0.0056 for comparisons between patients with missing and non-missing data on all three scores, *P*<0.0050 for comparisons between patients with missing and non-missing data on mortality, and at *P*<0.0167 for pairwise testing in comparative analyses of triage and prognostication scores.^32^ Test characteristics of sensitivity, specificity, positive predictive value (PPV), negative predictive value (NPV), positive likelihood ratio (PLR), and negative likelihood ratio (NLR) with associated 95% CIs were calculated for the three scores of interest.

## RESULTS

### Study Population

**Figure 1:**
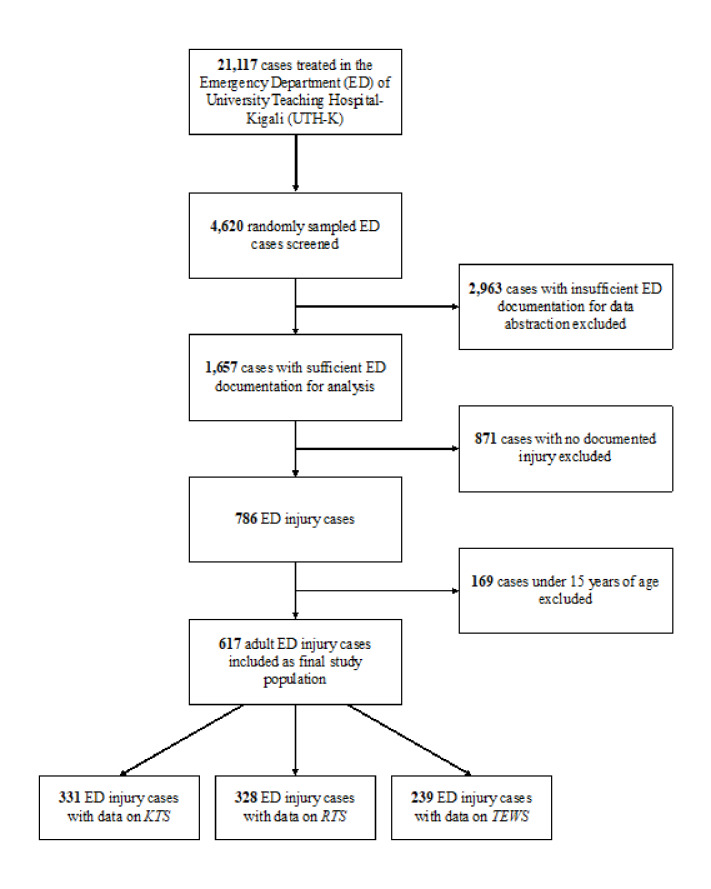
Study flow diagram. *KTS*, Kampala Trauma Score; *RTS*, Revised Trauma Score; *TEWS*, Triage Early Warning Scale.

Among 21,117 cases treated at the CHUK ED during the study period, 4620 were randomly screened for analysis. Data were gathered from 1657 cases, of which 617 were seeking care for injuries and included for analysis (Figure 1). For double-entered records, inter-rater reliability was excellent (κ = 0.95, standard error = 0.04). The majority of patients were male (72.5%) and the mean age was 32 years (IQR: 26–45). The most common anatomical regions of injury were craniofacial (39.3%), followed by lower extremity (38.7%) and upper extremity (23.0%). The most prevalent mechanisms of injury were road traffic accident (56.2%) and blunt injury or fall (21.9%). Fracture (46.0%) and contusion (12.0%) were the most common injury patterns. Approximately half of cases were admitted for inpatient care (52.8%). Among these patients, surgical intervention was performed on 74.8%, with open reduction being the most common procedure. Mortality through 14 days was 2.6%, and overall facility-based mortality was 3.4% (Table 1).

**Figure 2:**
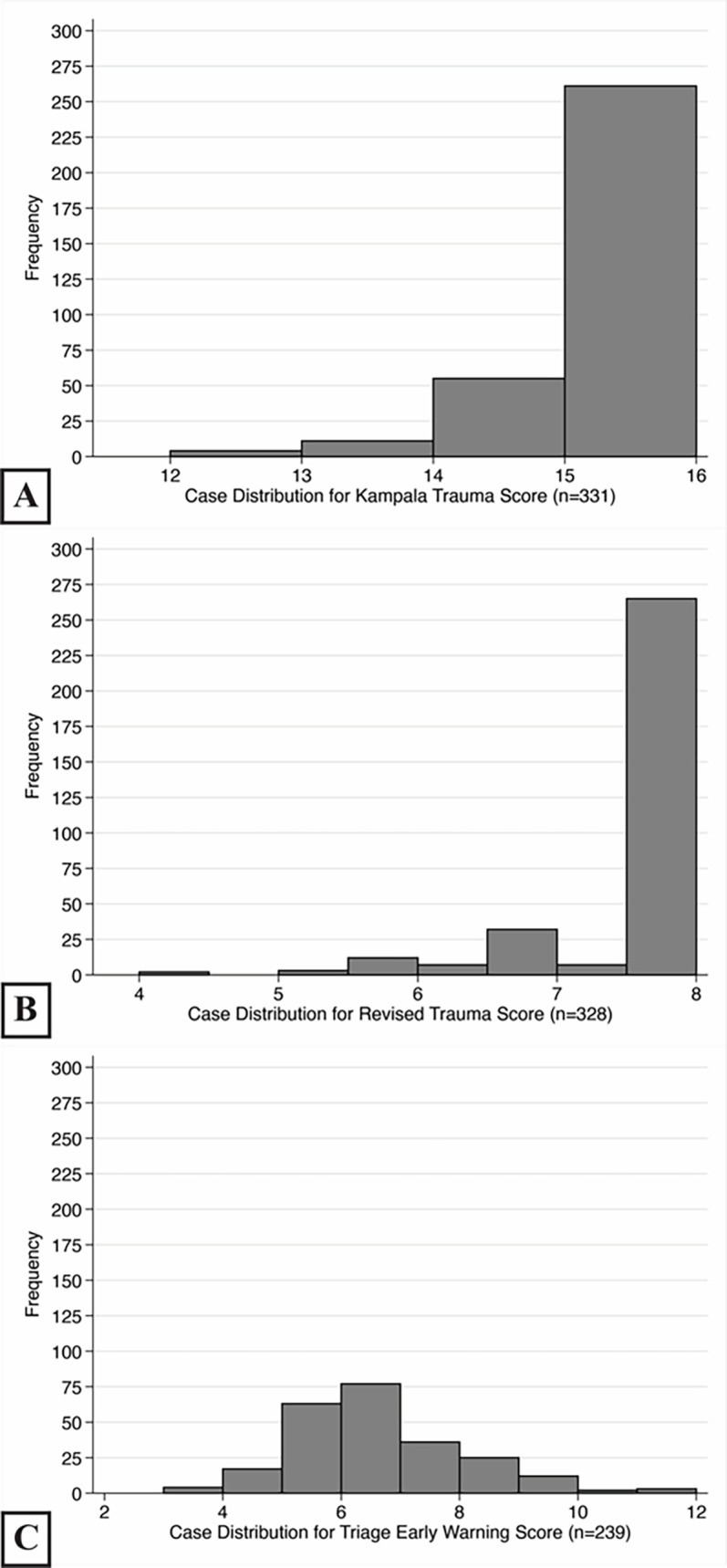
Histogram of score distributions.

Sufficient data was available to calculate KTS for 331 patients (53.6%), RTS for 328 patients (53.2%), and TEWS for 239 patients (38.7%). The most common missing measurements were respiratory rate for KTS and RTS (35.7%), and temperature for TEWS (43.4%). Among cases, 237 (38.4%) had complete data on all three scores. Patients had a mean score of 15.1 for KTS (median = 15, range: 12–16; Figure 2A), 7.6 for RTS (median = 7.8, range: 5.0–7.8; Figure 2B), and 6.2 for TEWS (median = 6, range: 3–12; Figure 2C). Patients with and without data on all three scores had no significant differences in age, gender, Glasgow Coma Scale (GCS), mechanism of injury, 14-day survival or overall survival based on the a priori threshold for multiple testing (Appendix 2). However, respiratory rate differed between patients with and without data (median = 18 vs 20 breaths per minute, *P*<0.001). Similarly, only respiratory rate (median = 20 vs 18 breaths per minute, *P*<0.001) and the KTS (median =15 vs 16, *P* = 0.001) differed between patients with and without data on mortality (Appendix 3).

### Prognostication Accuracy for 14-Day Mortality

**Figure 3:**
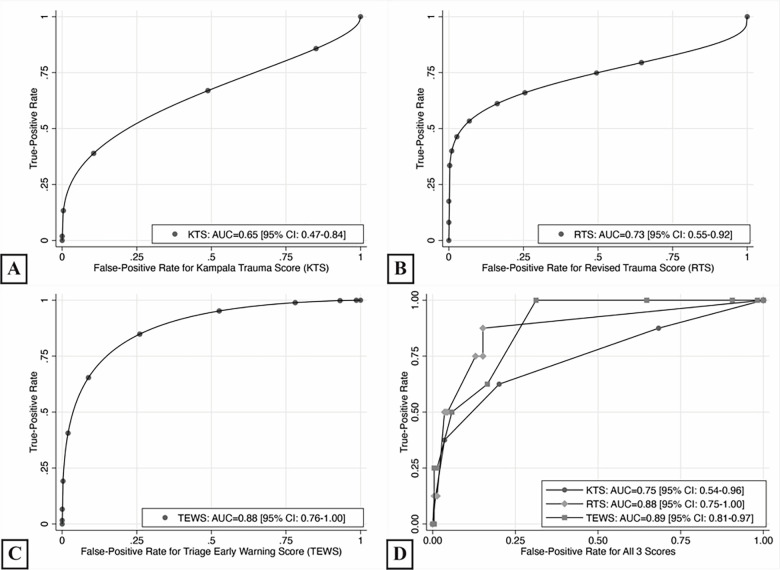
Diagnostic accuracy based on receiver operating curves for 14-day mortality. *KTS*, Kampala Trauma Score; *RTS*, Revised Trauma Score; *TEWS*, Triage Early Warning Scale.

For 14-day mortality, the TEWS had the highest discriminatory accuracy (AUC = 0.88, 95% CI, 0.76–1.00, *P*<0.001), followed by RTS (AUC = 0.73, 95% CI, 0.55–0.92, *P* = 0.013), with both scores performing significantly better than chance (Figure 3A–B). KTS had the lowest discriminatory accuracy (AUC = 0.65, 95% CI, 0.47–0.84, *P* = 0.108) and did not perform better than chance (Figure 3C). In comparative analysis, the TEWS had the most accurate diagnostic performance (AUC = 0.90), followed by the RTS (AUC = 0.84) and then the KTS (AUC = 0.75; Figure 3D). The RTS had significantly better discrimination than KTS (*P* = 0.011). No significant differences in performance were found in comparing the TEWS to the KTS (*P* = 0.058) or the RTS to the TEWS (*P* = 0.864).

### Prognostication Accuracy for Overall Facility-Based Mortality

**Figure 4:**
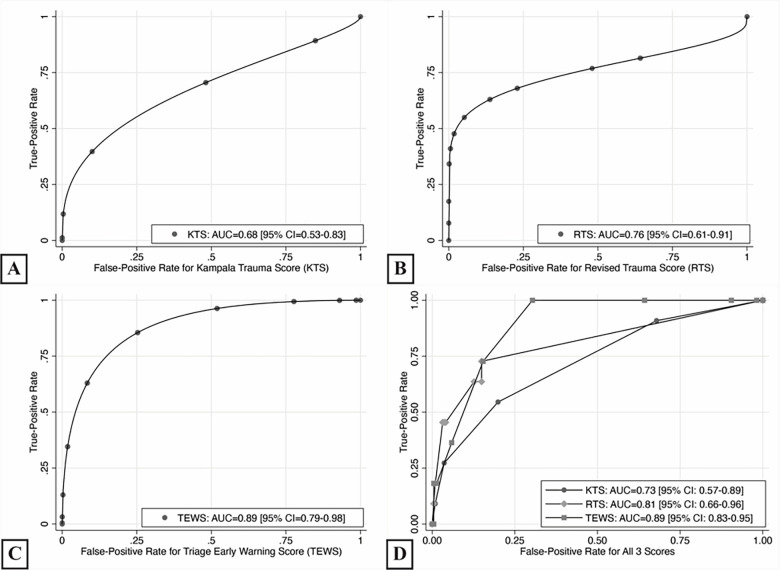
Diagnostic accuracy based on receiver operating curves for overall facility-based mortality. *KTS*, Kampala Trauma Score; *RTS*, Revised Trauma Score; *TEWS*, Triage Early Warning Scale.

For overall facility-based mortality, the TEWS had the highest discriminatory accuracy (AUC = 0.89, 95% CI, 0.79–0.98, *P*<0.001; Figure 4A), followed by RTS (AUC = 0.76, 95% CI, 0.61–0.91, *P*<0.001; Figure 4B) then KTS (AUC = 0.68, 95% CI, 0.53–0.83, *P* = 0.020; Figure 4C), with all three scores performing significantly better than chance. The TEWS had higher discriminatory accuracy for pairwise comparisons than the KTS (*P* = 0.007), but not the RTS (*P* = 0.207; Figure 4D). The KTS and the RTS did not have any significant differences (*P* = 0.101).

### Test Characteristics of Scores

Table 2 shows the range of test characteristics for TEWS using different cutoff points. Sensitivity and specificity were maximized at a threshold of ≥7 at 1.00 and 0.69, respectively. At a TEWS ≥9, the PLR demonstrated moderate clinical utility (8.65, 95% CI: 3.62–20.68). Appendix 4 shows the range of test characteristics for the KTS, and Appendix 5 shows the range of test characteristics for the RTS.

## DISCUSSION

This study evaluated the comparative accuracy of the KTS, RTS, and TEWS in predicting mortality following presentation for emergent injury care among adults in Rwanda. For the overall sample population, the TEWS exhibited the highest discriminatory accuracy among the three scores in predicting 14-day mortality and overall facility-based mortality. The TEWS also demonstrated significantly higher performance in predicting facility-based mortality compared to the KTS. These findings suggest that the TEWS may be the most clinically useful tool for risk-stratifying injured patients in the studied setting. The addition of trauma-specific scores, such as the KTS or RTS, may not yield additional clinical utility pertaining to mortality prognostication among ED patients seeking injury care.

Only two prior studies have compared the performance of the TEWS or SATS to trauma-specific scores. One study compared the KTS and TEWS for patients with gunshot wounds presenting to an urban hospital in South Africa.^23^ While the KTS had better diagnostic performance for mortality than the TEWS, as quantified by AUC, the difference was not statistically significant. The disparate accuracy results for the KTS vs the TEWS in the findings from the South Africa data as compared to the current data may be due to case selection in that the cohort looked at only a specific subset of injured patients, whereas the present study from Rwanda looked at injured patients more broadly. Another report comparing the SATS, KTS, and RTS in injury cases presenting to a tertiary hospital in Ghana also found no significant differences between the three scores in predicting mortality.^22^ Although the report from Ghana assessed the SATS, this triage approach uses the TEWS as a primary component in categorizing illness severity. The high performance of the TEWS in predicting injury mortality found in the Rwanda setting, coupled to the lack of benefit with trauma-specific scores from the Ghana cohort, supports the TEWS being a useful risk-stratification tool for trauma in and of itself, which has been suggested in prior studies.^23^,^33^,^34^ Nevertheless, further prospective evaluation in emergency care settings of this finding to more robustly validate the utility of the TEWS for the purpose of LMIC injury populations would be beneficial.

There are several potential explanations for the TEWS having the highest risk-stratification accuracy for trauma mortality in the present study population. The TEWS is a composite of physiological measurements, presence of trauma, and patient mobility. Several earlier studies have suggested that prognostication scores based purely on physiological measurements, such as the RTS, may be suboptimal for risk-stratifying injured patients due to certain trauma cases not necessarily presenting with physiological decompensation or varying levels of injury presenting with similar physiological measurements.^5^,^23^ While the KTS and TEWS both have scoring components accounting for the presence of trauma, the TEWS also uniquely has a scoring component representing patient functional status, in the form of mobility,^17^ which may have further improved the triaging of injured patients. Additionally, certain scoring components may be measured more accurately than others, resulting in better discrimination of illness states. For example, several studies have demonstrated heterogeneous levels of understanding and scoring for the GCS, a component of the RTS.^35^–^38^ In contrast, the TEWS uses a simplified alert, verbal, pain, and unresponsive scale to assess a patient’s level of consciousness, which is inherently a less complex differentiation than the GCS.

The present results also elucidate potential shortcomings in the inclusion of trauma-specific scores, such as the KTS and RTS, as additional triage tools for injured patients in LMIC ED settings. Earlier studies have contended that the use of separate triage tools for different presentations, such as medical or trauma patients, may introduce challenges including the necessary training of healthcare workers to apply an additional tool in practice and potential errors when applying separate metrics.^23^,^39^ Approximately 43% of CHUK ED admissions have medical presentations and the documented proportion of medical admissions for EDs in a similar setting have varied from 56–64%.^17^,^19^,^40^ Accordingly, an advantage of the TEWS over trauma-specific scores is its ability to be applied uniformly across both medical and trauma cases. Moreover, as the TEWS score can be integrated into the SATS tiered categories to guide the rapidity of needed injury interventions based on acuity, it may have greater clinical application than the KTS or RTS, which have no established cutoff points to inform decision-making for care provision.^22^ These factors, in addition to the greater relative prognostic accuracy, may support the use of general clinical care triage assessment tools, such as the TEWS, for risk-stratifying injured patients over the use of separate trauma prognostication scores.

Although the TEWS and the associated SATS have been successfully applied to predict hospitalization needs and mortality in several settings across sub-Saharan Africa,^14^,^16^,^19^–^21^ as well as outside sub-Saharan Africa,^6^,^41^ the score’s utility to inform clinical decision-making for acute injury care is an area in need of additional evaluation. This is highlighted by the calculated test characteristics for the population studied. Specifically, the PLRs derived from the TEWS only began to approach clinically useful values (ie. those that would substantially impact the post-test mortality probability) at a threshold of ≥ 9. Furthermore, although potentially clinically useful sensitivities were found at specific thresholds, these findings may be inaccurate, stemming from low numbers of mortality events in the lower score strata evaluated. This indicates that there may be opportunities for improvement of the TEWS to enhance clinical utility at specific threshold values.

Conversely, it is reasonable that emergency care practitioners may have the ability to appropriately risk-stratify patients independent of the use of formal triage or prognostication scores, but such clinical acumen would likely exist as a continuum based on providers’ experience levels and training. Future prospective research, designed and appropriately powered to evaluate the incremental clinical utility of the TEWS and other risk-stratification scores in injured patients as compared to provider gestalt, is needed to better inform training, resource utilization and emergency care globally.

## LIMITATIONS

There are limitations to the present study. First, due to the retrospective nature of the data, information was missing information for some cases. This may have introduced bias in the results, despite this study’s use of rigorous methods, including double-entering of records and random sampling of analyzed cases. However, a comparison of characteristics between the cases in the study population with and without data for the primary predictive analysis found no statistically significant differences in variables except for a two-breaths-per-minute difference in respiratory rate, which is unlikely a clinically significant difference. Missing data for mortality may have also introduced bias into the study. While a comparison of cases with and without data on mortality found no differences for most variables, it did find a similar two-breaths-per-minute difference for respiratory rate and one point in median KTS scores.

Second, due to lack of follow-up data, all analyses operated on the assumption of survival if a patient was discharged. As a result, the present study population’s mortality may be under-reported, and the comparative performance of the three scores may differ with the inclusion of deaths following discharge. However, for triage of emergently injured patients, there is still considerable clinical utility in risk-stratifying death during admission. Third, standardized use of TEWS at the CHUK during the study period may have affected the performance of this score, which impacted the course of care, relative to the KTS and RTS.^14^ However, the TEWS’ higher relative accuracy in discriminating mortality in the study population, despite these patients ostensibly receiving more urgent treatment due to identification at triage, may lend further credence to its validity in identifying the highest-risk injured patients.

Finally, due to the dataset being drawn from the ED of a single, tertiary care institution these results may not be generalizable to all settings, especially those with fewer resources. However, the present findings represent evidence in the comparative accuracy of trauma scores and generalized triage scores in predicting mortality following injury, which may form the basis of future comparative work and guide improvements in injury care in similar settings.

## CONCLUSION

Among a cohort of injured ED patients seeking care in the Rwanda study setting, the TEWS had the highest prognostic accuracy for 14-day and overall facility-based mortality, compared to KTS and RTS. This is one of the first studies comparing the TEWS to injury-specific scores globally and the first from Rwanda. The results from this population and earlier comparisons suggest that the addition of an injury-specific score in the triage of injured patients in LMICs may offer little advantage beyond standard triage approaches for mortality prognostication. However, given the retrospective nature of the data, further prospective research is needed to understand the most optimal triage and prognostication approaches for injured ED patients in LMICs.

## Supplementary Information











## Figures and Tables

**Table 1:** Summary characteristics for study population.

Variable	Number (%) or Median (IQR)
Gender	
Male	447 (72.4%)
Female	169 (27.4%)
Missing	1 (0.2%)
Age (Years)	32 (26–45)
Heart rate	85 (72–98)
Respiratory rate	19 (18–20)
Systolic blood pressure	124 (112–135)
Glasgow Coma Scale	
3–8	21 (3.4%)
9–12	40 (6.5%)
13–15	364 (59.0%)
Missing	192 (31.1%)
Anatomical regions of injuries*	
Craniofacial	243 (39.3%)
Thorax	96 (15.6%)
Abdomen or pelvis	89 (14.4%)
Neck or spine	51 (8.3%)
Upper extremity	142 (23.0%)
Lower extremity	239 (38.7%)
Other	34 (5.5%)
Types of Injuries*	
Fracture	284 (46.0%)
Classified as open	105 (17.0%)
Burn	12 (1.9%)
Contusion	74 (12.0%)
Dislocation	33 (5.3%)
Site of injury	
Home	41 (6.7%)
Work site	162 (26.2%)
Street	159 (25.8%)
Health center	8 (1.3%)
Other or unknown	247 (40.0%)
Transport by formal prehospital services	
Yes	437 (70.8%)
No	180 (29.2%)
Mechanism of injury	
Road traffic accident	347 (56.2%)
Blunt injury or fall	135 (21.9%)
Penetrating injury	75 (12.2%)
Burn	12 (1.9%)
Animal encounter	5 (0.8%)
Unknown	43 (7.0%)
ED disposition	
Admitted	326 (52.8%)
Discharged to home	137 (22.2%)
Transferred	9 (1.5%)
Death	4 (0.7%)
Unknown	141 (22.8%)
Emergency department length of stay (Days)	1 (0–2)
Inpatient disposition (n = 326)	
Discharged to home	273 (83.7%)
Transferred	34 (10.4%)
Death	17 (5.2%)
Unknown	2 (0.7%)
Inpatient length of stay (Days)	7 (3–16)
Received surgical intervention	
Yes	244 (74.8%)
No	82 (25.2%)
Surgical Interventions Performed*	
Open reduction	94 (28.8%)
Wound debridement	66 (20.2%)
Closed reduction with external fixation	49 (15.0%)
Craniotomy	37 (11.3%)
Laparotomy	25 (7.7%)
Other	51 (15.6%)
Overall Length of Stay (Days)	6 (2–14)
14-Day Survival	
Alive	462 (74.9%)
Dead	16 (2.6%)
Unknown	139 (22.5%)
Overall Facility-Based Survival	
Alive	457 (74.1%)
Dead	21 (3.4%)
Unknown	139 (22.5%)

* Percentages do not add up to 100% for anatomical region of injuries, types of injuries, and surgical interventions performed because categories were non-mutually exclusive for these variables.

*IQR*, interquartile range.

**Table 2:** Test characteristics for triage early warning score for 14-day mortality outcome.

Threshold Score	Number (%)	Sensitivity [95% CI]	Specificity [95% CI]	PPV [95% CI]	NPV [95% CI]	PLR [95% CI]	NLR [95% CI]
≥6	154 (66.1%)	1.00 [1.00–1.00]	0.35 [0.29–0.41]	0.05 [0.02–0.09]	1.00 [1.00–1.00]	1.54 [1.40–1.70]	0.00 [0.00-0.00]
≥7	78 (33.5%)	1.00 [1.00–1.00]	0.69 [0.63–0.75]	0.10 [0.04–0.17]	1.00 [1.00–1.00]	3.21 [2.65–3.90]	0.00 [0.00-0.00]
≥8	42 (18.0%)	0.63 [0.29–0.96]	0.84 [0.79–0.88]	0.12 [0.02–0.22]	0.98 [0.97–1.00]	3.80 [2.06–7.01]	0.45 [0.18–1.10]
≥9	17 (7.3%)	0.50 [0.15–0.85]	0.94 [0.91–0.97]	0.24 [0.03–0.44]	0.98 [0.96–1.00]	8.65 [3.62–20.68]	0.53 [0.27–1.06]

*PPV*, positive predictive value; *NPV*, negative predictive value; *PLR*, positive likelihood ratio; *NLR*, negative likelihood ratio.
