# Supplementary material for: Performance of Prognostication Scores for Mortality in Injured Patients in Rwanda
Source: West J Emerg Med. 2021 Jan 22;22(2):435–44. doi: 10.5811/westjem.2020.10.48434 (PMC7972380; doi:10.5811/westjem.2020.10.48434)
Supplement: Supplementary file 1 [file wjem-22-435-s001.docx]

**Appendix 1:** Description of Prognostication Scores

| **Score Parameter** | **Kampala Trauma Score (Range: 5–16)** | **Revised Trauma Score (Range: 0–7.84)** | **Triage Early Warning Score (Range: 0–17)** |
| --- | --- | --- | --- |
| Age (years) | **1:** <5 or >5  **2:** 5–55 |  |  |
| Systolic blood pressure (mm Hg) | **1:** Undetectable  **2:** 1–49  **3:** 50–89  **4:** >89 | **0:** 0  **1:** 1–49  **2:** 50–75  **3:** 76–89  **4:** >89  *Score component is multiplied by 0.7326* | **0:** 101–199  **1:** 81–100  **2:** 71–80 or >199  **3:** <71 |
| Respiratory rate (breaths per minute) | **1:** <9  **2:** >30  **3:** 10–29 | **0:** 0  **1:** 1–5  **2:** 6–9  **3:** >29  **4:** 10–29  *Score component is multiplied by 0.2908* | **0:** 9–14  **1:** 15–20  **2:** <9 or 21–29  **3:** >29 |
| Heart rate (beats/minute) |  |  | **0:** 51–100  **1:** 41–50 or 101–110  **2:** <41 or 111–129  **3:** >129 |
| Temperature (° Celsius) |  |  | **0:** 35–38.4°  **2:** <35° or >38.4° |
| AVPU (Alert, Verbal, Pain, Unresponsive) scale | **1:** Unresponsive  **2:** Responsive to painful stimuli  **3:** Responsive to verbal stimuli  **4:** Alert |  | **0:** Alert  **1:** Responsive to verbal stimuli  **2:** Responsive to painful stimuli or confused  **3:** Unresponsive |
| Glasgow Coma Scale (GCS) |  | **0:** GCS 3  **1:** GCS 4–5  **2:** GCS 6–8  **3:** GCS 9–12  **4:** GCS 13–15  *Score component is multiplied by 0.9368* |  |
| Presence of trauma | **1:** ≥2 serious injuries  **2:** 1 serious injury  **3:** 0 serious injuries |  | **0:** Trauma not present  **1:** Trauma present |
| Mobility |  |  | **0:** Walking  **1:** Walking with help  **2:** On a stretcher or immobile |
