# Supplementary material for: Performance of Prognostication Scores for Mortality in Injured Patients in Rwanda
Source: West J Emerg Med. 2021 Jan 22;22(2):435–44. doi: 10.5811/westjem.2020.10.48434 (PMC7972380; doi:10.5811/westjem.2020.10.48434)
Supplement: Supplementary file 2 [file wjem-22-435-s002.docx]

**Appendix 2:** Differences in Characteristics for Patients With and Without Data for All Three Scores

| **Variable** | **Number (%) or Median (IQR) for Patients With Data for All 3 Scores**  **(n=237)** | **Number (%) or Median (IQR) for Patients Without Data for All 3 Scores**  **(n=380)** | ***P*-Value** |
| --- | --- | --- | --- |
| Gender |  |  | 0.042 |
| Male | 183 (77.2%) | 264 (69.7%) |  |
| Female | 54 (22.8%) | 115 (30.3%) |  |
| Age (Years) | 32 (26–45) | 32 (25–44) | 0.716 |
| Heart Rate | 85 (71–100) | 84 (75–96) | 0.927 |
| Respiratory Rate | 20 (18–20) | 18 (16–20) | <0.001 |
| Systolic Blood Pressure | 124 (112–136) | 125 (113–135) | 0.723 |
| Glasgow Coma Scale |  |  | 0.987 |
| 3-8 | 12 (5.1%) | 9 (4.8%) |  |
| 9-12 | 22 (9.3%) | 18 (9.6%) |  |
| 13-15 | 203 (85.7%) | 161 (85.6%) |  |
| Mechanism of Injury |  |  | 0.769 |
| Road Traffic Accident | 145 (62.2%) | 202 (59.2%) |  |
| Blunt Injury or Fall | 49 (21.0%) | 86 (25.2%) |  |
| Penetrating Injury | 31 (13.3%) | 44 (12.9%) |  |
| Burn | 6 (2.6%) | 6 (1.8%) |  |
| Animal Encounter | 2 (0.9%) | 3 (0.9%) |  |
| 14-Day Survival |  |  | 0.905 |
| Alive | 224 (96.6%) | 238 (96.8%) |  |
| Dead | 8 (3.4%) | 8 (3.2%) |  |
| Overall Facilities-Based Survival |  |  | 0.825 |
| Alive | 221 (95.3%) | 236 (95.9%) |  |
| Dead | 11 (4.7%) | 10 (4.1%) |  |
