# Supplementary material for: Performance of Prognostication Scores for Mortality in Injured Patients in Rwanda
Source: West J Emerg Med. 2021 Jan 22;22(2):435–44. doi: 10.5811/westjem.2020.10.48434 (PMC7972380; doi:10.5811/westjem.2020.10.48434)
Supplement: Supplementary file 3 [file wjem-22-435-s003.docx]

**Appendix 3:** Differences in Characteristics for Patients With and Without Data on Mortality

| **Variable** | **Number (%) or Median (IQR) for Patients With Data on Mortality**  **(n=478)** | **Number (%) or Median (IQR) for Patients Without Data on Mortality**  **(n=139)** | ***P*-Value** |
| --- | --- | --- | --- |
| Gender |  |  | 0.370 |
| Male | 351 (73.4%) | 96 (69.6%) |  |
| Female | 127 (26.6%) | 42 (30.4%) |  |
| Age (Years) | 32 (26–46) | 31 (25–41) | 0.180 |
| Heart Rate | 85 (72–99) | 83 (75–89) | 0.252 |
| Respiratory Rate | 20 (18–20) | 18 (16–20) | 0.001 |
| Systolic Blood Pressure | 124 (111–135) | 129 (118–139) | 0.026 |
| Glasgow Coma Scale |  |  | 0.338 |
| 3-8 | 21 (5.3%) | 0 (0.0%) |  |
| 9-12 | 39 (9.8%) | 1 (4.4%) |  |
| 13-15 | 340 (85.0%) | 22 (96.6%) |  |
| Mechanism of Injury |  |  | 0.053 |
| Road Traffic Accident | 281 (61.9%) | 65 (55.6%) |  |
| Blunt Injury or Fall | 107 (23.6%) | 27 (23.1%) |  |
| Penetrating Injury | 53 (11.7%) | 21 (18.0%) |  |
| Burn | 11 (2.4%) | 1 (0.9%) |  |
| Animal Encounter | 2 (0.4%) | 3 (2.6%) |  |
| Kampala Trauma Score | 15 (15–16) | 16 (16–16) | 0.001 |
| Revised Trauma Score | 7.84 (7.84–7.84) | 7.84 (7.84–7.84) | 0.227 |
| Triage Early Warning Score | 6 (5–7) | 5 (5–5) | 0.041 |
