# Supplementary material for: Performance of Prognostication Scores for Mortality in Injured Patients in Rwanda
Source: West J Emerg Med. 2021 Jan 22;22(2):435–44. doi: 10.5811/westjem.2020.10.48434 (PMC7972380; doi:10.5811/westjem.2020.10.48434)
Supplement: Supplementary file 4 [file wjem-22-435-s004.docx]

**Appendix 4:** Test Characteristics for Kampala Trauma Score for 14-Day Mortality Outcome

| **Threshold Score** | **Number (%)** | **Sensitivity [95% CI]** | **Specificity [95% CI]** | **PPV**  **[95% CI]** | **NPV**  **[95% CI]** | **PLR**  **[95% CI]** | **NLR**  **[95% CI]** |
| --- | --- | --- | --- | --- | --- | --- | --- |
| ≤15 | 217 (67.2%) | 0.67  [0.40–0.93] | 0.33  [0.28–0.38] | 0.04  [0.01–0.06] | 0.96  [0.93–1.00] | 0.99  [0.66–1.49] | 1.02 [0.45–2.30] |
| ≤14 | 70  (21.7%) | 0.50  [0.22–0.78] | 0.79  [0.75–0.84] | 0.09  [0.02–0.15] | 0.98  [0.96–1.00] | 2.43  [1.32-4.46] | 0.63 [0.36-1.11] |
| ≤13 | 15  (4.2%) | 0.25  [0.01–0.50] | 0.96  [0.94–0.98] | 0.20  [0.00–0.40] | 0.97  [0.95–0.99] | 6.48  [2.10–19.98] | 0.78  [0.56–1.08] |

PPV = positive predictive values, NPV = negative predictive values, PLR = positive likelihood ratio, NLR = negative likelihood ratio
