# Supplementary material for: Performance of Prognostication Scores for Mortality in Injured Patients in Rwanda
Source: West J Emerg Med. 2021 Jan 22;22(2):435–44. doi: 10.5811/westjem.2020.10.48434 (PMC7972380; doi:10.5811/westjem.2020.10.48434)
Supplement: Supplementary file 5 [file wjem-22-435-s005.docx]

**Appendix 5:** Test Characteristics for Revised Trauma Score for 14-Day Mortality Outcome

| **Threshold**  **Score** | **Number (%)** | **Sensitivity**  **[95% CI]** | **Specificity**  **[95% CI]** | **PPV**  **[95% CI]** | **NPV**  **[95% CI]** | **PLR**  **[95% CI]** | **NLR**  **[95% CI]** |
| --- | --- | --- | --- | --- | --- | --- | --- |
| ≤7 | 56  (17.4%) | 0.58  [0.30–0.86] | 0.84  [0.80–0.88] | 0.13  [0.04–0.21] | 0.98  [0.96–1.00] | 3.69  [2.14–6.35] | 0.49  [0.25–0.97] |
| ≤6 | 17  (5.3%) | 0.42  [0.14–0.70] | 0.96  [0.94–0.98] | 0.29  [0.08–0.51] | 0.98  [0.96–0.99] | 10.76  [4.51–25.68] | 0.61  [0.38–0.98] |

PPV = positive predictive values, NPV = negative predictive values, PLR = positive likelihood ratio, NLR = negative likelihood ratio
